# Transactions of the American Medical Association

**Published:** 1878-05

**Authors:** 


*Transactions of the American Medical Association.* Volume XXVIII. 1878.

It is an unmistakable fact, that a progressive spirit actuates this Association. Unimportant contentions about legislative and other business matters are no longer suffered to excite agitation of the general assembly, or rival in interest the scientific work and purposes of the meeting. Greater perfection of organization has been attained by the experience of this Association, and of other similarly large bodies, which have held their sessions during the last few years. Work is more economically and better done by reference whenever possible to officers or committees selected for their competence for particular duties. The President, Henry I. Bowditch, M. D., in his address, in criticising the work of the Association, recommends the appointment by the Judicial Committee of a committee to solicit scientific communications, and the reference of the proceedings of Sections to a committee of experts, unknown, with power as to publication of the same. Increasing scientific enthusiasm among the members of the Association and in the Sections will accomplish much in time, and may make the scientific character of the meetings as prominent as the social has been in previous history.

Of much that is of value in the volume, especially interesting to us have been the address by James P. White, M. D., as chairman of the Section of Obstetrics, and the paper by Gilman Kimball, M. D., on Extirpation of the Uterus. Other noticeable articles which may be mentioned, besides addresses of chairmen of sections, are: Suspension as a means of treating Spinal Disorders, by Benj. Lee, A.M., M.D.; Medio-Bilateral Lithotomy, by W. T. Briggs, M.D.; Stricture from Masturbation, by L. W. Gross, A.M., M.D.; Tuberculosis in Milch Cows, and the Contagiousness of Tuberculosis by the Digestive Organs, by A. N. Bell, M.D., Etiology of Enteric Fever, by J, L. C. Cabell, M.D.; Recognition and Management of the Gouty state in Diseases of the Skin, by L. D. Bulkley, A.M., M.D.; etc.

